# WHO GETS HOSPICE VISITS AT THE END OF LIFE: A LOOK AT MEDICARE'S HOSPICE SERVICE INTENSITY ADD-ON POLICY

**DOI:** 10.1093/geroni/igac059.2513

**Published:** 2022-12-20

**Authors:** Michael Plotzke, Thomas Christian

**Affiliations:** Abt Associates, Saint Louis, Missouri, United States; Abt Associates, Cambridge, Massachusetts, United States

## Abstract

In 2016, the Centers for Medicare & Medicaid Services (CMS) implemented the Service Intensity Add-on (SIA) payment for the Medicare Hospice Benefit. The SIA incentivizes direct patient care by registered nurses and medical social workers during the last seven days of life through additional payments to hospices. We examined 100% of Fee-for-Service Medicare hospice claims in Fiscal Year 2020 to determine whether SIA utilization varies by patient and hospice characteristics. Using hospice level fixed-effects regression, we examine the associations between SIA utilization and various beneficiary and hospice characteristics. Relative to beneficiaries with cancer as a principal diagnosis, we find that other diagnoses are associated with only a slight reduction in SIA utilization (a reduction between 0.6 and 1.1 percentage points - where the average SIA utilization overall is equal to 85.3%). Those identifying as black had a reduction in SIA of 1.3 percentage points compared to those identifying as white. Compared to a patient dying on a Sunday, a patient’s death on Tuesday through Friday was associated with a higher likelihood of SIA (between 9.7 and 11.6 percentage points more likely) and more SIA minutes (between 29.9 minutes and 36 minutes). All reported results were statistically significant at the 0.1% level. Based on these results, we find that SIA utilization varied by certain characteristics. As a result, CMS should continue to monitor rates of SIA utilization to better understand whether any patient groups appear to be underserved at the end of life.

